# Mindfulness, rumination, and coping skills in young women with Eating Disorders: A comparative study with healthy controls

**DOI:** 10.1371/journal.pone.0213985

**Published:** 2019-03-15

**Authors:** Ana Hernando, Raquel Pallás, Ausiàs Cebolla, Javier García-Campayo, Claire J. Hoogendoorn, Juan Francisco Roy

**Affiliations:** 1 Department of Psychology and Sociology, University of Zaragoza, Zaragoza, Spain; 2 Eating Disorders Unit, Hospital Clínico Universitario, SALUD, Zaragoza, Spain; 3 Departament de Personalitat, Avaluació i Tractaments Psicològics, Universitat de València, CIBEROBN, Fisiopatologia de la Obesidad y la Nutrición, Valencia, Spain; 4 Instituto de Investigación Sanitaria Aragón, Hospital Universitario Miguel Servet, Zaragoza, Spain; 5 Red de Actividades Preventivas y Promoción de la Salud en Atención Primaria (RediAPP), Zaragoza, Spain; 6 Ferkauf Graduate School of Psychology, Yeshiva University, Bronx, New York, United States of America; 7 Facultad de Educación y Salud, Universidad Camilo José Cela, Madrid, Spain; Universitat de Barcelona, SPAIN

## Abstract

Eating Disorders (ED) have been associated with dysfunctional coping strategies, such as rumination. Promoting alternative ways of experiencing mental events, based on a mindfulness approach, might be the clue for learning more effective coping and regulatory strategies among young women with ED. This study examined the comparison between patients with ED diagnosis and healthy subjects in mindfulness, rumination and effective coping. In addition, we analyzed the independent association of those with the presence of ED. The study sample was formed by two groups of young women ranged 13–21 years: Twenty-five with an ED diagnosis and 25 healthy subjects. They were assessed by using the Freiburg Mindfulness Inventory (FMI) and the Responses Styles Questionnaire (RSQ). Our findings show that ED patients have significantly lesser average scores in mindfulness and effective coping than the healthy sample (p < .05). Also, our data concludes that mindfulness and effective coping independently predict the presence or absence of ED in young women. The study results suggest that training mindfulness abilities may contribute to making effective coping strategies more likely to occur in ED patients, which is incompatible with some eating-related symptoms. Further studies are needed, trough prospective and experimental designs, to evaluate clinical outcomes of mindfulness training among young women with ED.

## Introduction

In the last decades, the ideal of thinness has become not only a sign of beauty but also of success and self-control [1]. At the same time, prevalence rates of eating disorders (ED) show a two- to five times increase in the past three decades as [2] described using current data in Spain [3, 4]. Similarly, Yeo and Hughes [5] documented that ED are the third most common pathology in adolescent females after obesity and asthma. EDs can also have severe medical and psychological consequences [2, 6, 7]; for example, anorexia nervosa is associated with the highest mortality rate of any psychiatric disorder [7–9]. Thus, it is both essential and urgent to investigate cognitive and behavioral aspects of ED and develop effective treatments to alleviate its symptoms.

Fairburn holds that EDs are in essence ‘cognitive disorders’ [10]. Some disordered eating-related behaviors are driven by negative self-evaluations, emotions or thoughts [11]. For example, patients with anorexia nervosa and bulimia nervosa often experience negative emotion states [6, 12], and focus repetitively on those experiences and its consequences, which is a maladaptive coping strategy called rumination [13, 14]. This ruminative thinking seems to share characteristics such as ‘inflexible’ and ‘difficult to control behavior’, proposed by Deci and Ryan [15], to define automatic behaviors. Nevertheless, Rawal, Park, and Williams [16] suggested that rumination may be a relevant feature in the information processing in ED, dealing with negative consequences as psychological distress and the maintenance of abnormal eating [17]. In addition, recent studies show the importance of rumination as a vulnerability factor for disordered eating, and in the aggravation of ED, since it is a coping strategy to distress related to negative affect, maladaptive behavior and psychological distress [18, 19].

Mindfulness is known for helping individuals to disengage from automatic thoughts, dysfunctional thinking, habits, and unhealthy behaviors patterns [11, 20]. Mindfulness has been defined as open receptivity to the present and includes acceptance of the current moment, focused attention and awareness, as well as the metacognitive ability to take distance from one’s cognitions [20]. Deci and Ryan [15] explained that automatic or ‘mindless’ behaviors resist changes, particularly when motives or processes remain out of awareness. And, according to Brown and Ryan [20], attention and awareness are crucial notions in their definition of mindfulness, considered both as an ‘inherent state of consciousness’ and as a disposition (trait) that vary both inter- and intra-individuals. Attention and awareness are particularly important to avoid rumination and thus to improve self-regulation, with individual differences in the frequency of mindful states over time being inversely related to rumination behavior [17, 20]. Lattimore, Mead, Irwin, Grice, Carson, and Malinowski [21] also provided evidence that supports the relevancy of interoceptive awareness, in the relationship between dispositional mindfulness and psychological problems associated with development and maintenance of ED. Additionally, Brown and Ryan [20] pointed to rumination as an example of blunting consciousness and its negative consequences for well-being; while mindfulness was related to positive well-being and less cognitive and emotional disturbance.

Several studies have examined the importance of mindfulness training and other metacognitive skills in relation to ED, showing the individual alternative ways of experiencing mental events, particularly taking distance from them, or dis-identification, that involves becoming aware of one’s mental functioning and taking distance from it, which is believed that dysfunctional coping strategies are less likely to occur [11, 17, 22, 23]. Elices et al [24] found that individuals with ED, compared to healthy controls, engage to a lesser extent in a direct experience while eating; but they did not include potential psychopathological confounders as rumination or coping in their analyses. Additionally, the study conducted by Masuda, Price, Anderson, and Wendell [25] showed the importance of how a person responds to events, and its role in the development and maintenance of negative psychological outcomes, supporting the premises of mindfulness-based interventions. Alberts *et al*., [11] suggested that mindfulness could help to reduce food cravings, body image concern, dichotomous thinking or avoidance-based coping strategies. For these reasons, mindfulness training could be a useful intervention to reduce rumination, and increase effective coping skills, though first, the role of trait mindfulness needs to be better understood.

Based on previous findings, we aimed to investigate in a clinical context if rumination, effective coping and mindfulness differ among young women with an ED diagnose and healthy controls. Furthermore, we tested the hypothesis that mindfulness, rumination and effective coping are significant and independent predictors of ED and significantly differ between those subsamples.

## Materials and methods

This research has been evaluated and approved by the "Comité Ético de Investigación Clínica de Aragón" (CEICA, C.I. PI17/0364). Every individual entering the study received information about privacy data norms and regulations (Law 15/1999) [26]. In addition, this study fulfilled Spanish national regulations regarding patients’ autonomy and rights and biomedical research, including the possibility to revoke their consent and to drop out the study when any participant would desire (Law 41/2002 and Law 14/2007) [27, 28].

Finally, subjects were asked to sign two copies of the consent as they accept to provide information and complete the measures. Participants were provided with instructions before assessment, which included information about the instructions of both tests and how they must be completed.

### Participants

This study arises from a collaborative study between the University of Zaragoza and the ED Unit at the *Hospital Clínico Universitario* (HCU), both at Zaragoza. Aragón (Spain). The sample consisted of n = 50 participants. This size was in line with the criterion suggested by Freeman whereby the number of participants must be ≥10 (k+1), with k being the number of co-variables [29].

### Clinical subsample

Twenty-five consecutive patients were recruited from the ED Unit at the *HCU*. Inclusion criteria were as follows: consultation patients (1) aged 21 years or younger (2) female and (3) diagnosed with ED (AN, BN or OSFED). Exclusion criteria were as follows: (1) diagnosis or Obesity or ED during childhood, (2) other mental disorder and (3) currently or recently pregnant.

### Healthy controls subsample

Participants in this group were randomly selected from the Zaragoza University College or high school student population. A total of 25 healthy female subjects were recruited to be matched with the clinical subsample. Inclusion criteria were as follows: (1) aged 21 years or younger and (2) female, and exclusion criteria were (1) ED diagnosis or problematic eating behavior, and (2) other mental disorders.

### Measures

Subjects in both groups completed the same two self-rating questionnaires. Mindfulness was assessed using the short form *Freiburg Mindfulness Inventory* (FMI) [30]. This is a 14-item scale is considered robust, with a high (r = .95) correlation with the 30-item version and high reliability (alpha = .86). Moreover, the FMI has been considered to measure the core of the mindful-ness construct and was designed to measure mindfulness in subjects with or without meditation experience [30]. The short form of the FMI has been validated in Spanish by Pérez-Verduzco and Laca-Arocena (2017) [31]. Rumination and effective coping were measured using a modified, actualized version [32] of the original *Response Styles Questionnaire* (RSQ) [33]; a measure with good psychometric indicators (i.e.: Rumination, Cronbach’s alpha = .90; and effective coping, Cronbach’s alpha = .83) which is used to assess individuals’ tendencies, to adopt different ways of responding to their negative emotions. In addition, study subjects were asked about their current age and studies.

### Procedure

All patients completed the questionnaires in the ED Unit at the HCU under clinical and research supervision. Duration to complete all the data was not fixed, but no subject spent more than 30 minutes to fulfill all the questionnaires. Healthy young women completed those in their homes under the study researchers supervision. In both groups, subjects had at their disposal only a pen, a table and a chair to complete the questionnaires and exactly the same contextual conditions; while responding the questionnaires, subjects were only in the company of the supervisor, who was located out of the subjects’ visual field with the purpose of not disturbing or distracting the study participants.

### Statistical analyses

All analyses were carried out using the Statistical Package for the Social Sciences (SPSS) version 20.0. Before starting these analyses reversed items were transformed to direct scores in the FMI. In every single independent analysis, the null hypothesis was rejected if p ≤ 0.05.

Prior to data analyses, outliers were detected through a detailed review of every questionnaire. Student’s t-test was used to test the first hypothesis. We previously checked if both subsamples fulfilled the assumptions to calculate the Student’s t-test. Firstly, we calculated if both subsamples followed a normal distribution. This normality assumption was tested using this two normally test: Shapiro-Wilk and Kolmogorov-Smirnov. Secondly, we tested if both subsamples had the same variance through the Levene’s test. Odds ratios estimations were calculated through hierarchical bivariate and multivariate logistic regression analyses, in order to test differences in the risk of ED, when compared with healthy subjects, according to rumination, effective coping and mindfulness, related to the presence or absence in those illnesses.

## Results and discussion

Our original sample was composed of 50 young women. A detailed review of every questionnaire was carried out prior to data analyses. One patient was excluded from final analyses because as the Field Study Record confirmed, she filled the questionnaires after a long evaluation at the ED Unit, so she was most probably cognitively exhausted. Information regarding scores on the scales refers to the whole sample, and indicate descriptive statistics for mindfulness (mean = 35.43; *SD =* 5.76), rumination (mean = 52.12; *SD* = 9.75) and effective coping (mean = 45.12; *SD =* 10.41). The study sample was composed of two subsamples:

In the clinical subsample the mean age was 16.6 (*SD* = 2.24); all were students of bachelor’s degree (24%) or secondary education (76%), and according to DSM-IV-TR criteria [31] they were diagnosed as having: *307*.*5* EDNOS (44%), *307*.*1* AN-R (52%) and *307*.*51* BN (4%).

Healthy controls showed a mean age of 19.08 years *(SD* = 0.64, range = 18–21). All of the participants are students of bachelor’s degree (92%) or secondary education (8%).

Comparative statistics and Student’s *t-*tests results and descriptive statistics are shown in Table 1. The descriptive analyses show the mean and the standard deviation of both groups regarding the three variables. Student’s *t-*tests were conducted to compare means of both groups across the three variables representing metacognitive processes: mindfulness ability, rumination and effective coping skills. Results showed that mindfulness and effective coping means were significantly lower in the clinical subsample (Student´s t = .015 and .007, respectively), while rumination mean was marginally higher in that group.

**Table 1:** Comparison of means and independent samples t-test in mindfulness, rumination and effective coping between patients and healthy women groups.

	ED		NO ED		*t*-test	Sig.	95% CI	
	M	SD	M	SD				
**Mindfulness**	33.42	6.17	37.36	4.68	-2.53	.015	-7.08	-0.80
**Rumination**	54.88	11.02	49.48	7.67	1.99	.052	-0.04	10.83
**Effective coping**	41.13	9.95	48.96	9.51	-2.82	.007	-13.42	-2.42

According to data shown in Table 1, the clinical subsample mean ($\bar{x}$ = 33.42) was statistically significantly lower in mindfulness, when compared to the healthy subsample ($\bar{x}$ = 37.36). Moreover, the clinical subsample mean in effective coping ($\bar{x}$ = 41.12) was also significantly lower when compared to the healthy controls ($\bar{x}$ = 48.95). In contrast, the average score in rumination of the clinical subsample was higher than healthy subsample, although this difference was marginally significant.

In addition, bivariate Binary Logistic Regression (LR) was conducted for ED risk estimation through variables based on the four predictor variables: age, mindfulness, rumination and effective coping.

Before a multivariate binary logistic regression study would be conducted, we tested if age fulfilled appropriate statistical assumptions, because OR was suspiciously high and age only ranged from 13 to 21. For instance, normality test (using *Kolmogorov-Smirnov test* and *Shapiro-Wilk*) were carried out for age, showing that this variable did not follow a normal distribution (*p<0*.*001* in both normality tests). In addition, *Levene’s test* showed that age did not share the same variance in the two groups of ED (*F = 44*.*125; p<0*.*001)*. Thus, we considered that age was skewing these specific results, so this variable was excluded from further LR analyses. Due to evident and model-guided theoretical reasons, we additionally hypothesized that the three factors mindfulness, rumination, and effective coping were high related to each other, most probably due to sharing a common cognitive construct. Thus, a Correlation analysis was conducted.

On one hand, results showed medium-high correlations between mindfulness and effective coping (r = -0.38), while on the other hand, those variables showed a low correlation with rumination (mindfulness: r = 0.10; effective coping: r = -0.01). To avoid confounding between these factors, we conducted multivariate analysis in two separate models in order to exclude both biases (age abnormal distribution and mindfulness and effective coping high correlation). Hierarchical multivariate LR analysis results are shown in Table 2.

**Table 2:** Hierarchical multivariate logistic regression of metacognitive skills by the presence of ED.

		OR		95,0% C.I.		Sig
**Unadjusted**		Age	4.28	1.58	11.59	.004
		Mindfulness	1.14	1.02	1.28	.021
		Rumination	0.94	0.88	1.00	.060
		Effective coping	1.09	1.02	1.16	.013
**Adjusted**	**Model 1**	Mindfulness Rumination	1.12 0.95	1.00 0.88	1.26 1.02	.044 .146
	**Model 2**	Effective coping Rumination	1.08 0.95	1.01 0.88	1.15 1.01	.021 .120

As shown in Table 2, multivariate LR models showed that mindfulness and effective coping independently predicted the presence or absence of ED in the study sample. LR analyses indicate that every increased score in mindfulness (FMI has a maximum score of 30) increases 12% the risk of ED and 8% in the case of rumination.

## Conclusions

To our knowledge, his is the first study that compares mindfulness, effective coping and rumination in ED female patients compared to healthy women. Our findings demonstrate that young patients diagnosed with ED show significantly lower levels of mindfulness (p = 0.015) and effective coping (p = 0.007) when compared to a group of healthy women. Specifically, our study supports the hypothesis that patients diagnosed with ED are more likely to engage in dysfunctional coping strategies, which has been previously associated with disordered eating behaviors [13, 16, 22]. Consistent with previous findings [11, 15, 17, 21], our study provides new data supporting the fact that individuals who exhibit higher levels of awareness and attention to one’s thoughts, may be less likely to engage in a disordered eating-related behavior, highlighting the role of mindfulness in self-regulatory and metacognitive processes. This position concurs with Brown and Ryan [20], who found that mindfulness, involving awareness of one’s behavior, was related to and predicted more positive well-being and less cognitive disturbance.

In contrast, our data do not reach strictly significant differences in rumination between patients with ED and healthy participants (p = 0.052). This data in relation to rumination doesn’t allow us to make certain conclusions about whether rumination is clearly related to ED or not. However, past research does support this association [13, 17, 18, 20]. This lack of association may be due to are small sample size, and larger studies are needed to confirm whether rumination plays a role.

Bearing in mind both statements, a mindfulness and effective coping focused interventional approach, would be potentially more effective in treating ED, than directing main intervention aims at rumination. In sum, mindfulness practice could be a helpful resource to create new mental landscape and behaviors to deal with different situations (emotions, reactions…) in these patients, bringing up alternative and more effective coping responses [11, 13, 18, 21, 22]. Furthermore, clinical research innovations are suggested by our study data, as including new questionnaires related to disordered eating-related behavior (as EAT-40 for example), in this sense, we could define better clinical and healthy subsamples. Likewise, clinical subsample could be differentiated by specific diagnosis (AN, BN, EDNOS) in further studies. Moreover, those studies should also include other questionnaires as the Spanish version of MAAS (Mindful Attention Awareness Scale; Soler et al) [34] or the FFMQ (Five Facets of Mindfulness Questionnaire; Cebolla et al) [35]; and finally, comparison with men subsamples could contribute with more data about self-regulatory processes in general [23] and its relationship with ED development, course, treatment and prognosis.

There were also limitations in the present study; firstly, questionnaires were completed in exams period (June). Considering that our sample were college students, factors related to this situation (anxiety, stress, fatigue…) could skew our results; thus, administrate questionnaires in another period of the academic year could be an adequate alternative. Secondly, there is a difference of 2.48 years between the mean age of each subsample (clinical and healthy subjects). Despite it is relatively frequent to see both adolescents and young women included together in ED-related articles [24], the potential bias produced by the age difference in our results has to be considered.

Another limitation of our study is related to the small sample size, but the study subject’s characteristics and inclusion/exclusion criteria are very specific (only young women aged 14-21years, clinical subsample with an ED diagnose), and recent and similar studies in which clinical ED patients are compared to healthy controls, such as Elices et al [24], also use similar sample sizes. Moreover, our study is based on a cross-sectional design, which implies that causal inferences cannot be made. Further studies with a higher sample size, comparable age groups in both subsamples and a prospective longitudinal approach, could permit test new hypotheses concerning the role of these factors over time. This would allow us to better understand the role of rumination include other protective or risk factors to develop problematic eating behavior or ED [17, 22].

Despite these limitations, our study contributes towards a promising emerging clinical perspective exploring the investigation of mindfulness in eating behavior domain [11]. In this line, recent studies have appeared, focusing on different but related characteristics as impulsivity and interoceptive awareness [21]; or ruminative thinking although by using only clinical or non-clinical samples [19, 18]. Moreover, our findings suggesting that mindfulness is associated to a lesser presence of an EDs diagnosis, point out that mindfulness intervention studies are to be developed in order to test the mid- to long-term efficacy of mindfulness training on ED outcomes [16, 19, 22, 23]. If those potential trials would show positive results, the quality of life and prognosis of individuals with disordered eating-related cognitions, behaviors or disorders, could be substantially improved.

## Supporting information

S1 Dataset (SAV)

## References

[1] Perpiñá C. Trastornos alimentarios In: Belloch A, Sandín B, Ramos F, editors. Manual de psicopatología. Madrid; McGraw-Hill; 2008 p. 403–421.

[2] Peláez MA, Labrador FJ, Raich RM. Prevalencia de los trastornos de la conducta alimentaria: consideraciones metodológicas. Intern Jour Psych Psychol Therapy. 2005;5(2):135–148.

[3] Instituto Nacional de la Salud. Protocolo de trastornos del comportamiento alimentario. Madrid: INSALUD 1995.

[4] American Psychiatric Association. Diagnostic and statistical manual of mental disorders (DSM-5©).Washington (D.C): American Psychiatric Pub; 2013.

[5] Yeo M, Hughes E. Eating Disorders. Early identification in general practice. Aust Fam Physician. 2011; 40 (3): 108–111. 21597510

[6] Raich RM. Anorexia, bulimia y otros trastornos alimentarios. Madrid: Pirámide; 2011.

[7] Kaye WH. Neurobiology of Anorexia and Bulimia Nervosa. Physiol Behav. 2008; 94(1): 121–135. 10.1016/j.physbeh.2007.11.037 18164737 PMC2601682

[8] Garcés E. Anorexia nerviosa: una mirada relacional. Revista de Trabajo y Salud. 2005; 51: 101–132.

[9] Kaye WH, Fudge J, Paulus M. New insights into symptoms and neurocircuit function of anorexia nervosa. Nat Rev Neurosci. 2009; 10: 573–584. 10.1038/nrn2682 19603056 PMC13038070

[10] Fairburn CG. Cognitive Behavior Therapy and Eating Disorders. New York: The Guilford Press; 2008.

[11] Alberts HJ, Thewissen R, Raes L. Dealing with problematic eating behaviour. The effects of a mindfulness-based intervention on eating behaviour, food cravings, dichotomous thinking and body image concern. Appetite. 2012; 58: 847–857. 10.1016/j.appet.2012.01.009 22265753

[12] Keel P. Eating Disorders. New Jersey: Pearson Prentice Hall; 2005.

[13] Aldao A, Nolen-Hoeksema S. Specificity of cognitive emotion regulation strategies: A transdiagnostic examination. Behav Res Ther. 2010; 48: 974–983. 10.1016/j.brat.2010.06.002 20591413

[14] Nolen-Hoeksema S. The role of rumination in depressive disorders and mixed Anxiety/Depressive symptoms. J Abnorm Psychol. 2000; 109(3): 504–11. 11016119

[15] Deci EL, Ryan RM. Self-determination theory: When mind mediates behavior. The Journal of Mind and Behavior. 1980; 1: 33–43.

[16] Rawal A, Park RJ, Williams MG. Rumination, experiential avoidance, and dysfunctional thinking in eating disorders. Behav Res Ther. 2010; 48: 851–859. 10.1016/j.brat.2010.05.009 20598670 PMC2923742

[17] Cowdrey FA, Park RJ. The role of experiential avoidance, rumination, and mindfulness in eating disorders. Eat Behav. 2012; 13:100–105. 10.1016/j.eatbeh.2012.01.001 22365790

[18] Maraldo TM, Zhou W, Dowling J, Vander Wal JS. Replication and extension of the dual pathway model of disordered eating: The role of fear of negative evaluation, suggestibility, rumination and self-compassion. Eat Behav. 2016;23:187–194. 10.1016/j.eatbeh.2016.10.008 27816857

[19] Naumann E, Tuschen-Caffier B, Voderholzer U, Schäfer J. Svaldi J. Effects of emotional acceptance and rumination on media-induced body dissatisfaction in anorexia and bulimia nervosa. J Psychiatr Res. 2016;82:119–125. 10.1016/j.jpsychires.2016.07.021 27491068

[20] Brown KW, Ryan RM. The benefits of being present: mindfulness and its role in psychological well-being. J Pers Soc Psychol. 2003;84(4):822 12703651 10.1037/0022-3514.84.4.822

[21] Lattimore P, Mead BR, Irwin L, Grice L, Carson R, Malinowski P. ‘I can’t accept that feeling’: Relationships between interoceptive awareness, mindfulness and eating disorder symptoms in females with, and at-risk of an eating disorder. Psychiatry Res. 2017;247:163–171. 10.1016/j.psychres.2016.11.022 27915166

[22] Masuda A, Wendell JW. Mindfulness mediates the relation between disordered eating-related cognitions and psychological distress. Eat Behav. 2010;11:293–296. 10.1016/j.eatbeh.2010.07.001 20850066

[23] Lavender JM, Jardin BF, Anderson DA. Bulimic symptoms in undergraduate men and women: Contributions of mindfulness and thought suppression. Eat Disord. 2009;10:228–231. 10.1016/j.eatbeh.2009.07.002 19778752

[24] Elices Matilde, et al Direct experience while eating: Laboratory outcomes among individuals with eating disorders versus healthy controls. Eating behaviors. 2017,27:23–26. 10.1016/j.eatbeh.2017.10.002 29111496

[25] Masuda A, Price M, Anderson P, Wendell JW. Disordered eating-related cognition and psychological flexibility as predictors of psychological health among college students. Behav Modif. 2010;34: 3–15. 10.1177/0145445509351569 20051522

[26] Jefatura del Estado. Ley de Protección de Datos de Carácter Personal. [Ley 15 de 1999]. DO: BOE-A-1999-23750. [Internet] 1999 December [cited 2012 June 10] Available from: http://www.boe.es/buscar/doc.php?id=BOE-A-1999-23750

[27] Jefatura del Estado. Ley básica reguladora de la autonomía del paciente y de derechos y obligaciones en materia de información y documentación clínica. Ley 41 de 2002. DO: BOE-A-2002-22188. [Internet] 2002 November [cited 2012 June 10] Available from: http://www.boe.es/buscar/doc.php?id=BOE-A-2002-22188

[28] Jefatura del Estado. Ley de Investigación biomédica. DO: BOE-A-2007-12945. [Internet] 2007 July [cited 2012 June 10]. Available from: http://www.boe.es/buscar/doc.php?id=BOE-A-2007-12945

[29] Freeman DH. Applied categorical data analysis. New York: Marcel Dekker; 1978.

[30] Walach H, Buchhleld N, Buttenmüller V, Kleinknwcht N, Schmidt S. Measuring Mindfulness—The Freiburg Mindfulness Inventory (FMI). Pers Individ Dif. 2006;40:1543–1555.

[31] Pérez-Verduzco G and Laca-Arocena FA. Traducción y validación de la versión abreviada del Freiburg Mindfulness Inventory (FMI-14). Revista Evaluar. 2017; 17(1):80–93.

[32] Knowles R, Tai S, Christensen I, Bentall R. Coping with depression and vulnerability to mania: a factor analytic study of the Nolen-Hoeksema (1991) Response Style-Questionnaire. Br J Clin Psychol. 2005;44:99–112. 10.1348/014466504X20062 15826347

[33] Nolen-Hoeksema S. Responses to depression and their effects on the duration of depressive episodes. J Abnorm Psychol. 1991;100(4):569 1757671 10.1037//0021-843x.100.4.569

[34] Soler Ribaudi J, et al Propiedades psicométricas de la versión española de la escala Mindful Attention Awareness Scale (MAAS). Actas Esp Psiquiatr 2012; 40(1):18–25. 22344492

[35] Cebolla A, et al Psychometric properties of the Spanish validation of the Five Facets of Mindfulness Questionnaire (FFMQ). The European Journal of Psychiatry. 2012; 26(2):118–126.

